# Exploring the seasonal yield variability, production risk and efficiency: the case of rice farms in Bangladesh

**DOI:** 10.1016/j.heliyon.2022.e10559

**Published:** 2022-09-07

**Authors:** Md Abdus Salam

**Affiliations:** Department of Agricultural Economics, Bangabandhu Sheikh Mujibur Rahman Agricultural University, Gazipur, 1706, Bangladesh

**Keywords:** Yield variability, Production risk, Technical efficiency, True random effect model, Rice farm, Seasonal panel, Bangladesh

## Abstract

Farmers in developing countries face different rice production risks, influencing yield variability. This study investigates the technical efficiency (TE) and production risk of Boro and Aman rice in Bangladesh. A stochastic production frontier has been used assuming a true random effect (TRE) model with flexible risk properties using two-year seasonal plot-level panel data of 5088 observations for Boro rice and 5638 observation for Aman rice. The empirical result of the risk model shows that labor, fertilizer, seed, and farm capital have a significant risk-decreasing effect for Boro rice. In contrast, the cultivated area of rice and average mean temperature have a significant risk-increasing effect on Boro rice. Labor and pesticide have significant risk-decreasing effects, whereas cultivated area, fertilizer and seed has a significant risk-increasing effect on Aman rice production. The average TE was 76% and 72% for Boro and Aman rice, respectively. Results suggest a high degree of variability in TE estimates, and the average farmer could increase rice yield by 24% and 28% by improving technical management without increasing the existing inputs. Large farms are more technically efficient than other farm categories. It is also observed that small farms and medium farms significantly decrease technical efficiency for Boro rice while significantly increasing for Aman rice. Moreover, technical efficiency declined over time for Boro rice, while it improved for Aman rice in Bangladesh. Production risk, however, declined over time for both Boro and Aman rice.

## 1 Introduction

Rice production is inherently risky due to the heterogeneous production environment, which is the leading staple food and the major source of people's calorie intake in Bangladesh. Shelley et al. (2016) argued that the annual population growth rate is 2 million, and the total population will touch 238 million by 2050. Moreover, the environmental degradation and climate change with increasing population pressure, if the supply of rice is not in line with its demand, is reflected in staple food crises. In this situation, increasing rice yield is required to increase total rice production to feed this ever-increasing population. The ability to obtain the highest yields depends on a bundle of input factors, input allocation decisions, soil quality, environmental factors, etc. Some of the inputs contribute positively to obtaining maximum output while others may not or negatively influence. The agricultural sector faces many types of risks, while rice farmers face multiple challenges, including production and environmental risks that eventually affect a farmer's capability to attain the highest yield (Rizwan et al., 2020). Even if the household has access to these resources, the yield may fall severely (e.g., extreme weather, the incidence of pests, and diseases). Yang et al. (2016) argued that grain production is naturally risky due to the variability of biophysical factors like weather, diseases, and soil quality, resulting in increased yield variability. Therefore, how input factors affect yield variability (risk) is essential for input allocation decisions that are the risk related to the production technique and makes yield variability. Hence, the differences from the highest achievable yields to observed yields are unwanted. The input choices of farmers tend to affect the extent of output variability, although every farmer's goal is to employ the factors of inputs to obtain the maximum yield.

The appropriate input choice and distribution is an important research issue to minimize yield variability. Hence, it is a priority issue by policy-maker, researchers, and others to sustain growth and enhance potential rice productivity by reducing yield risk. Although, risk-averse farmers always try to mitigate uncertain risks through an appropriate choice of inputs. However, the application of inputs can either decrease or increase the variance of output level (production risk), but this effect is climatic, situational, and regional-specific (Just and Pope, 1978). Therefore, attempts should be made to increase the rice yield, and this may be a way to meet up the increasing demand for food in Bangladesh. The decision-making capability of farmers on inputs allocation is often affected by external variables which affect production risk that affects output supply (Villano and Fleming, 2006). Rice farming and harvesting are prone to failure due to the several uncertainty risks and pest and disease attacks in Bangladesh. Therefore, examining the association between the application of input and output variance would be helpful for producers through improving knowledge about the risk effects of their choice of inputs and risk management in rice farms for policy-makers. Among the three seasons for rice production in Bangladesh, Aman and Boro is the dominant season in terms of area and production for rice cultivation. This study focused on the factors affecting Aman and Boro rice's technical efficiency and production risk, respectively^1^.

Furthermore, suppose production risk has a significant impact on the decision of farmer's production. In that case, the effectiveness of the farmer's technical efficiency (TE) can vary significantly, suggesting that it is important to include production risk and farmer's response into the empirical approaches to estimate TE. Previous research has explored that on farms run by family members after they retire risk-averse, off-farm income reduces risk aversion (Picazo-Tadeo and Wall, 2011), while output variability is mainly caused by production risk (Tiedemann and Latacz-Lohmann, 2013). Labor and improved soil quality had significant risk-decreasing effects, while machinery applied significant risk-increasing effects on rice production in Central China (Yang et al., 2016). The environmental factor like moisture stress on dry season crops, rainfall, and extreme weather events were the most critical factors of production risk in rice-based farming systems (Kabir et al., 2019).

Many studies were conducted to estimate productivity and technical inefficiency in Bangladesh agriculture, including Gautam and Ahmed (2019), Azad and Rahman (2017), Bell et al. (2015), and Hasnain et al. (2015), where all the studies failed to estimate production risk in the production process. The findings of this study will deliver helpful evidence for all types of stakeholders involved in the design and implementation of programs and policies aimed at improving Boro and Aman rice production in Bangladesh. This study investigates and compares the micro-level evidence regarding magnitude and determinants that drive Boro and Aman rice yield variability. The main goal is how input levels and environmental and farm-specific features affect risk and inefficiency for achieving the highest potential yield of Boro and Aman rice production in Bangladesh using the True Random Effect (TRE) model analysis. Particular attention is paid to comparing yield variability between Boro and Aman rice production, factors that influence decreased or increased production risk and efficiency, and efficiency for farmers residing in different climate sub-regions due to variation in agro-ecological climate conditions and production practices.

## 2 Methodological approach and materials

The addition of heteroskedastic disturbance structure in the stochastic production frontier (SPF) model is applied to estimate rice farms' technical efficiency (TE), factors affecting TE, and the risk of rice production in Bangladesh. The SPF approach was originally developed by Aigner et al. (1977), and the general form of SPF is as follows

(1) $$Y_{it}=f\left(X_{it};\beta \right)\;exp\;\left(\varepsilon_{it}\equiv v_{it}-u_{it}\right)$$

Where, $Y_{it}$ is the quantity of rice harvested in plot i (i=1,2,3,4……N) in year t (t=1,2,3,4….T); the vector of inputs denoted by $X_{it}$. The parameter's vectors denote β to be calculated, and the error term $\varepsilon_{it}$, which is composed of two independent elements, $v_{it}$ and $u_{it}$. The term $v_{it}\;$ is assumed normally distributed errors that is symmetric identical and independent which represent random variation in output such as uncertainty of production with zero mean and variance $\sigma_{v}^{2}$ $\left[v_{it}∼\;N\left(0,\;\sigma_{v}^{2}\right)\right]$. This indicates that the factors are beyond the farmer's control, as are the effects of measurement errors and statistical noise. The non-negative random variable denotes $u_{it}$ $\left[u_{it}∼N^{+}\left(0,\sigma_{u}^{2}\right)\right]$ is related to the farmer's technical inefficiency assumed to be independent and identically distributed (iid) truncations of the half-normal distribution. The maximum likelihood method is used simultaneously to estimate the parameters of the SFP model, risk effect model, and inefficiency effect model for both Aman and Boro rice.

Many empirical studies on TE and production risk have been analyzed using a single structure with technical efficiency and production risk by applying a flexible production function representing the production technology. Following Yang et al. (2016), this study adopts the SFP model with a heteroscedasticity error framework which can be a positive or negative marginal effect of inputs on the risk of production that fulfills most of the Just and Pope's (1978) risk assumptions. The specification of the error in Eq. (1) takes the following form:

(2) $$\varepsilon_{it}=g\left(X_{it};\psi \right)\left[v_{it}-u_{it}\right]\;$$

where, the term $u_{it}s$ denotes the technical inefficiency $\left[u_{it}∼N^{+}\left(0,\sigma_{u}^{2}\right)\right]$ and independently distributed of the $v_{it}s$. By applying the specification in Eq. (2) and rewriting Eq. (1), we obtain the following Equation:

(3) $$Y_{it}=f\left(X_{it};\;\beta \right)+\;g\;\left(X_{it};\psi \right)v_{it}-g\left(Z_{it};\;\delta \right)u_{it}$$

Eq. (3) represents the specification of the SFP function with flexible risk properties (Villano and Fleming, 2006). The term $Y_{it}$ denotes observed rice production in plot i (i=1,2,3,4,……N) in year t (t=1,2,3,4….T). The term $f\left(X_{it};\;\beta \right)\;$ indicate the mean production function where input variables are $X_{it}$. The term $\beta_{it}$ indicates the vector of parameters of the mean production function to be estimated. The production risk function denotes $g\;\left(X_{it};\psi \right)v_{it}$, where $\;\psi_{it}$ represents the coefficient of the production risk function to be estimated. The term $g\left(Z_{it};\;\delta \right)u_{it}$ represent inefficiency effect function that captures the socioeconomic characteristics and technical inefficiency relationship. The term $Z_{it}$ denotes the vector of socioeconomic characteristics and $\delta_{it}$ represents unknown values of the technical inefficiency model. This study follows their clarification by identifying the mean and variance of production for the $i^{th}$ plot, and given the inputs values and the technical inefficiency effect (Equations (4) and (5)), $u_{it}$ as follows:

(4) $$E\left(Y_{it}\;|X_{it},\;u_{it}\right)=\;f\left(X_{it};\;\beta \right)-\;g\left(Z_{it};\delta \right)u_{it}\;$$

The production risk function or variance of production is defined as

(5) $$Var\;\left(Y_{it}\;|\;X_{it},\;u_{it}\right)=\;g^{2}\left(X_{it};\psi \right)$$

The marginal production risk concerning the $j^{th}$ input represents the partial derivative of the production risk concerning $X_{jt}$ that can be either negative or positive, are given below:

(6) $$\frac{\partial Var\;\left(Y_{it}\;|\;X_{it},\;u_{it}\right)}{\partial X_{ijt}}=\;\frac{\partial g^{2}\left(X_{it};\psi \right)}{\partial X_{ijt}}\;$$

where, $\frac{\partial g^{2}\left(X_{it};\psi \right)}{\partial X_{ijt}}\;<0\;$ indicates the risk decreasing of the $j^{th}\;$ input, and $\frac{\partial g^{2}\left(X_{it};\beta \right)}{\partial X_{ijt}}>0$ indicates the risk increases of the $j^{th}$ input for year t (Equation 6). The $i^{th}$ farm's output-oriented technical efficiency $\left(TE_{i}\right)$ in Eq. (7) denoted the ratio of observed production to the highest achievable production in the production frontier and expressed as follows:

(7) $$TE_{it}=\;\frac{E\left(Y_{it}\;|\;X_{it},\;u_{it}\right)}{E\;\left(Y_{it}\;|\;X_{it},\;u_{it}=0\right)}=\;\frac{f\left(X_{it};\;\beta \right)-\;g\;\left(X_{it};\psi \right)u_{it}}{f\left(X_{it};\;\beta \right)}=1-\;\frac{\;g\;\left(X_{it};\psi \right)u_{it}}{f\left(X_{it};\;\beta \right)}$$

where, the technical inefficiency ($TI_{it}$) is represented as $TI_{it}=\;\frac{\;g\;\left(X_{it};\;\psi \right)u_{it}}{f\left(X_{it};\;\beta \right)}$ . A one-step maximum likelihood (ML) technique is applied to estimate the parameters in Eq. (3) by optimizing the following log-likelihood function:

(8) $$lnL=constant-\frac{1}{2}\underset{it}{\sum }\text{ln}\left[\text{exp}\left(Z_{it};\delta \right)+\;\text{exp}\left(X_{it};\psi \right)\right]+\;\underset{it}{\sum }\ln\text{Φ}\frac{-\varepsilon_{it}\lambda_{it}}{\sqrt{\exp\left(Z_{it};\delta \right)+\exp\left(X_{it};\psi \right)}}\;-\;\frac{1}{2}\underset{it}{\sum }\frac{\varepsilon_{it}^{2}}{\exp\left(Z_{it};\delta \right)+\exp\left(X_{it};\psi \right)}$$

where, $\varepsilon_{it}=\;v_{it}-\;u_{it}$ corresponds to $\lambda_{it}=\;\sqrt{\exp\left(Z_{it};\delta \right)}/\sqrt{\exp\left(X_{it};\psi \right)}$; standard normal distribution denotes Φ (Equation 8). The TE of each rice farm can be measured over the restricted distribution of $u_{it}$ given $\varepsilon_{it}$ (Jondrow et al., 1982).

### 2.1 Specification and estimation of SFP model

Following similar studies on agriculture, e.g., Yang et al. (2016) and Onumah et al. (2018), this study specifies the translog stochastic production frontier to calculate production risk (PR) and TE. The time-invariant features of the farms may include some unobserved features such as specific innate capacity, which may not change over time. To address this problem, Greene (2005) proposed the "true" random-effects (TRE) model, which treats time-varying inefficiency and farm-specific time-invariant heterogeneity separately. This model deals with time variation in inefficiency while separating the time-varying inefficiency term from time-invariant unobserved heterogeneity. Following Greene (2005), this study adopts translog SPF of rice output for plot i in year t separately for Aman and Boro rice are as follows:

(9) $$ln\;\left(Y_{it}\right)=\;\beta_{0}+\;\overset{j}{\underset{j=1}{\sum }}\beta_{j}lnX_{ijt}+\frac{1}{2}\left(\overset{j}{\underset{j=1}{\sum }}\overset{k}{\underset{k=1}{\sum }}\beta_{jk}lnX_{ijt}lnX_{ikt}\right)+\gamma D_{it}+\alpha_{i}+v_{it}-u_{it}$$

Where, $Y_{it}$ is the quantity of rice harvested (kilogram) for plot i in year t. The term $X_{1}\ldots .X_{7}$ denotes the area of rice cultivation measured in decimal, total labor^2^ measured in work hours, the total cost for all types of fertilizer^3^, cost of seed, cost of pesticide^4^, cost of other inputs (including rental tools and machinery, draft animals, and irrigation (in cash)) and the farm capital^5^ for plot i, year t for Aman and Boro season, respectively. The term $v_{it}$ and $u_{it}$ are the same as those specified in Eq. (3). The term $\alpha_{i}$ denotes farm-specific time-invariant heterogeneity random variable that represents without specific distributional assumption. The specific distribution assumption for the error term of Greene's model is $v_{it}=\alpha_{i}+v{\text{′}}_{it}$ and $v{\text{′}}_{it}∼N\left(0,\sigma_{v}^{2}\right)$; $\alpha_{i}∼N\left(0,\sigma_{\alpha }^{2}\right)$ and the time-varying inefficiency term $u_{it}∼N^{+}(0,\sigma_{u}^{2}$). The composed error $\left(\varepsilon_{it}=v_{it}-u_{it}\right)$ that includes production risk, $g\left(X_{it};\psi \right)v_{it}$ and the technical inefficiency effect model, $q\left(Z_{it};\;\delta \right)u_{it}$ such that $\varepsilon_{it}=\;g\;\left(X_{it};\beta \right)v_{it}-q\left(Z_{it};\;\delta \right)u_{it}$ and the linear production risk model as follows:

(10) $$g\;\left(X_{it};\psi \right)v_{it}=\;\beta_{0}+\;\overset{k}{\underset{k=1}{\sum }}\psi_{k}X_{kit}$$

where, $X_{kit}\;$ denotes the seven input variables defined above, soil type (clay, loam, clay-loam, and sandy-loam), and environmental variable such as average monthly temperature and average monthly rainfall for Aman and Boro season, respectively. The term $\beta_{k}$ denotes risk parameters to be estimated. The technical inefficiency effect model is expressed as:

(11) $$q\left(Z_{it};\;\delta \right)u_{it}=\;\delta_{0}+\;\overset{j}{\underset{j=1}{\sum }}Z_{nit}\delta_{n}$$

The variables $Z_{nit}$ is the vector of household and farm features, region dummy that possibly affects the managerial capacity of farmers and $\delta_{n}$ represents the unknown parameter to be estimated. The parameters of the SFP model with flexible risk variables, i.e., Eqs. (9), (10), and (11), were calculated simultaneously in a one-stage applying maximum likelihood estimator for Aman and Boro rice separately.

### 2.2 Data description and summary statistics

**Figure 1:**
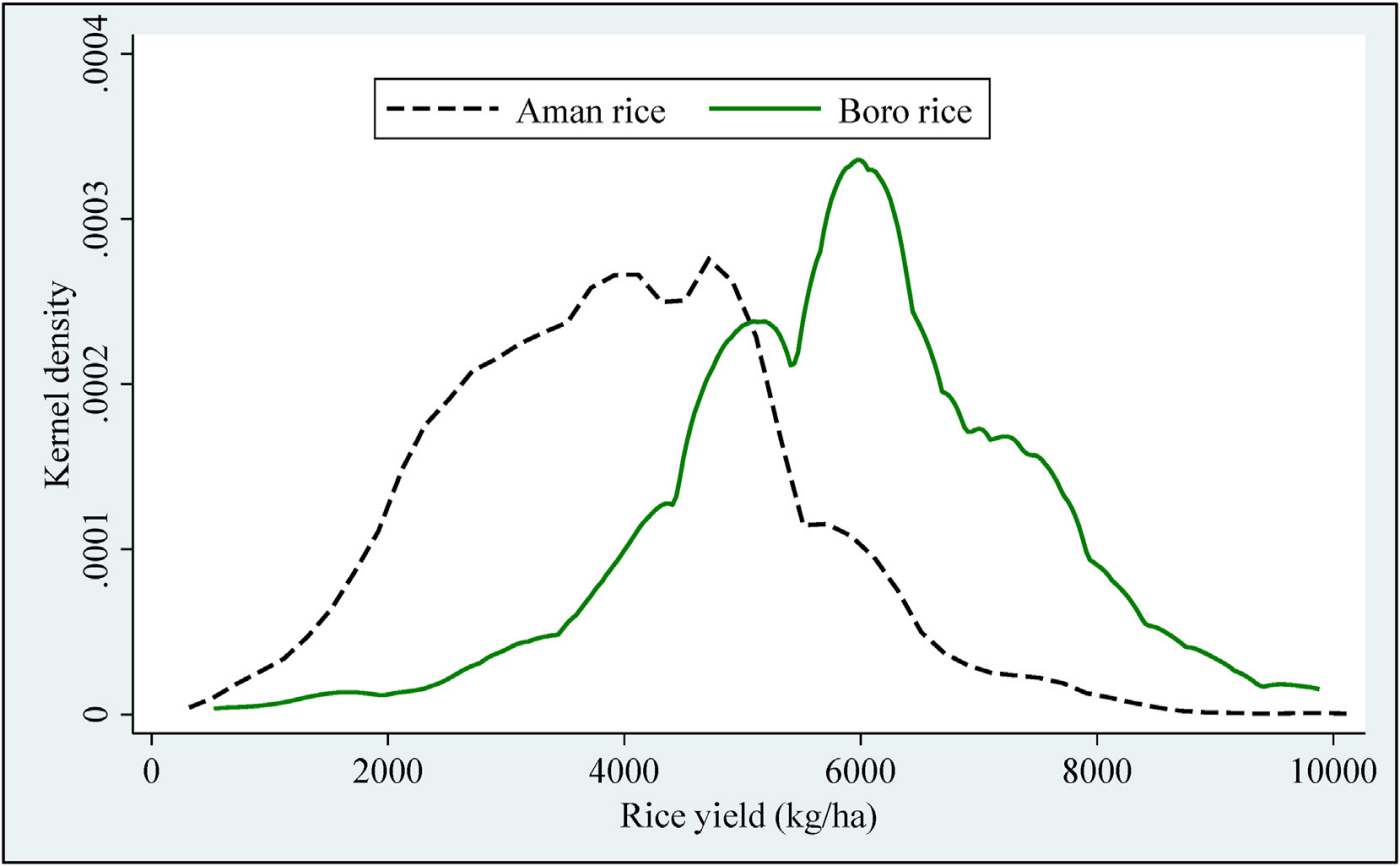
Distribution of yields for Aman and Boro rice for all plots.

This study uses second and third round nationally and statistically representative rural household data from Bangladesh Integrated Household Survey (BIHS). This survey was conducted from January to May 2015 (BIHS2015) and from November to May 2018–2019 (BIHS2018), covering the seven administrative divisions of the country under the supervision of researchers from the International Food Policy Research Institute (IFPRI. This study does not consider first-round data because farmers were asked only the main plot's information for inputs, and selecting main plots might cause serious bias in estimation results. Bell et al. (2015) explain the details for the first round. The total sample size of approximately 6,500 households in 325 primary sampling units (PSU) from seven administrative divisions of Bangladesh followed two stages of stratified sampling technique. This survey used the sampling frame based on the 2001 population census and later adjusted based on the latest 2011 Bangladesh population census. Plot-level information on various inputs and output quantities is collected using the interview schedule. This study considers only rice crops in different cropping seasons (Boro, Aman, and Aus^6^) in BISH2015 and BISH2018 due to available information in each plot. In BIHS2015, 3043 households cultivated rice in 13005 plots, of which Boro rice used 5737 plots and Aman rice used 6593 plots. On the other hand, In BISH2018, 3116 households cultivated rice in 12484 plots of land, of which the Boro rice used 5501 plots, and Aman rice used 6343 plots. The availability of input and output data for each crop planted on all plots allows the construction of plot-level seasonal panel datasets for Aman and Boro rice separately. To possibly control plot heterogeneity in stochastic production frontier (SPF) analysis, this study chose 2819 plots for Aman rice and 2544 plots for Boro rice grown in BISH2015 and BIHS2018, respectively. Finally, this study used 2819∗2 = 5638 observations for Aman and 2544∗2 = 5088 observations for Boro rice, respectively. The descriptive statistics of output and inputs variables, including the variables used in the risk model and inefficiency effect model, are presented in Table 1. In addition to input variables, this study considers soil type as a risk variable that captures the soil characteristics of production risk, and environmental factors such as rainfall and temperature that also influence rice production and rice production risk. Table 1 revealed that the average yield of rice is 5892 kg/ha and 3996 kg/ha for Boro and Aman rice in Bangladesh. Rice yield is highly variable in different seasons, ranging from 529 kg/ha to 9880 kg/ha and 549 kg/ha to 9880 kg/ha for Boro and Aman rice in Bangladesh. Figure 1 shows the visual inspection of Aman and Boro rice yield distribution. The summary also represents that the farmer used higher in all types of inputs except farm capital in Boro rice than Aman rice, of which fertilizer cost is much higher (43%) in Boro rice than Aman rice. The farmers in Boro rice production could be used advanced technology like hybrid variety, groundwater irrigation, etc., compared to Aman rice in Bangladesh. Summary statistics also show that farmers did not cultivate local varieties for Boro rice production. Groundwater is the primary source of irrigation for Boro rice production, while Aman rice mainly depends on rainfall, which could cause yield variability.

**Table 1:** Descriptive statistics of the relevant variables used in SFP model.

Variable	Description of the variable	Boro rice		Aman rice	
		Mean (S.D.)	Min - Max	Mean (S.D)	Min - Max
Yield	Rice output (kg/ha)	5892 (1518)	529–9880	3996 (1433)	549–9880
Land	Plot area (ha)	0.121 (0.148)	0.004–4.373	0.114 (0.116)	0.004–2.024
Labor	Quantity of both family and hired labor (hours/ha)	896.9 (438.5)	164.7–9711.7	792.1 (513.0)	35.3–16252.6
Fertilizer	Total cost for fertilizer (USD/ha)	133.9 (66.6)	0.0–960.6	93.6 (80.3)	0.0–3121.1
Seed	Total purchased and home supplied seed cost (USD/ha)	95.4 (44.5)	14.8–296.4	77.9 (41.9)	4.4–296.4
Pesticide	Total cost of pesticide, herbicide, insecticide (USD/ha)	29.7 (28.6)	0.0–741.0	25.0 (24.8)	0.0–254.1
Other input	Total cost of rental machinery, draft animals, irrigation (USD/ha)	329.1 (167.1)	0.0–3724.8	183.6 (129.6)	0.0–2675.9
Farm capital	Total current market value of own farm assets (USD/ha)	1892 (5699)	0.0–87873	1985 (6336)	0.0–114423
Local variety	1 if local variety is cultivated in the plot, 0 otherwise	-----	------	0.123 (0.328)	0–1
HYV	1 if HYV variety is cultivated in the plot, 0 otherwise	0.830 (0.375)	0–1	0.838 (0.369)	0–1
Hybrid	1 if hybrid variety is cultivated in the plot, 0 otherwise	0.170 (0.375)	0–1	0.040 (0.196)	0–1
Rain fed	1 if the plot is irrigated by rainfed, 0 otherwise	0.010 (0.101)	0–1	0.554 (0.497)	0–1
Ground water	1 if the plot is irrigated by groundwater, 0 otherwise	0.789 (0.408)	0–1	0.396 (0.489)	0–1
Surface water	1 if the plot is irrigated by surface water, 0 otherwise	0.201 (0.401)	0–1	0.049 (0.217)	0–1
*Inefficiency explaining variables*					
Landless	If the farm ownland is below 0.50 acres	0.348 (0.096)	0.08–0.490	0.358 (0.098)	0.08–0.495
Marginal	If the farm own land is between 0.51 to 1.0 acres	0.768 (0.136)	0.515–0.995	0.762 (0.135)	0.515–0.995
Small	If the farm own land is between 1.01 to 2.50 acres	1.597 (0.422)	1.0–2.495	1.634 (0.411)	1.0–2.495
Medium	If the farm own land is between 2.51 to 5.0 acres	3.464 (0.746)	2.50–5.00	3.420 (0.674)	2.50–5.00
Large	If the farm own land is greater than 5.00 acres	8.276 (3.78)	5.06–19.11	8.183 (3.95)	5.06–20.12
Head male	Dummy; 1 if the head is male; 0 otherwise	0.963 (0.188)	0–1	0.958 (0.200)	0–1
Education	Years of formal schooling completed by the household head	3.803 (4.072)	0–16	4.080 (4.122)	0–16
Family size	Number of persons in the household	5.403 (2.130)	1–23	5.371 (1.981)	1–19
Off-farm income	Total income from off-farm activities (USD)	1787 (1698)	0–20621	1737 (1644)	0–20621
Southeastern	1 if the plot is to the southeastern region, 0 otherwise	0.028 (0.165)	0–1	0.106 (0.308)	0–1
Northeastern	1 if the plot is to the northeastern zone, 0 otherwise	0.051 (0.219)	0–1	0.045 (0.207)	0–1
Northern part of north	1 if the plot is to the northern part of northern zone, 0 otherwise	0.058 (0.234)	0–1	0.080 (0.271)	0–1
Northwestern	1 if the plot is to the northwestern zone, 0 otherwise	0.127 (0.333)	0–1	0.134 (0.341)	0–1
Western	1 if the plot is to the western zone, 0 otherwise	0.133 (0.340)	0–1	0.110 (0.313)	0–1
Southwestern	1 if the plot is to the southwestern zone, 0 otherwise	0.142 (0.349)	0–1	0.221 (0.415)	0–1
South central	1 if the plot is to the south central zone, 0 otherwise	0.461 (0.499)	0–1	0.304 (0.460)	0–1
*Additional variable for risk model*					
Rainfall	The monthly mean rainfall (mm)	145.9 (36.5)	91.6–335.3	141.8 (31.8)	91.2–402.9
Temperature	Monthly average temperature (Celsius)	25.24 (0.60)	21.57–26.34	25.30 (0.58)	20.43–26.34
Sandy	1 for sandy soil, 0 otherwise	0.034 (0.182)	0–1	0.058 (0.234)	0–1
Clay	1 for clay soil, 0 otherwise	0.028 (0.164)	0–1	0.020 (0.141)	0–1
Loam	1 for loam soil, 0 otherwise	0.177 (0.381)	0–1	0.178 (0.383)	0–1
Clay-loam	1 for clay-loam soil, 0 otherwise	0.554 (0.497)	0–1	0.498 (0.500)	0–1
Sandy-loam	1 for sandy-loam soil, 0 otherwise	0.207 (0.405)	0–1	0.246 (0.431)	0–1
Observation		5088		5638	

Note: Standard deviations are in parentheses.

Among the variable used in the inefficiency function, the farm category^7^ shows that the mean farm size is similar between Boro and Aman rice. Aman rice producers have a higher average year of schooling, although below the primary level for both groups. Average family size, off-farm income, average monthly rainfall, temperature, etc., are slightly different between the production seasons. Farmers are trying to redistribute their time from the agricultural sector to the off-farm sector if they have the opportunity to get a higher return from off-farm. Participation of households in off-farm can significantly affect farm productivity and efficiency because off-farm income can facilitate farm management by purchasing flexible inputs and increasing information regarding farm inputs and technology through higher access to urban areas. In our sample, on average, the highest 46% and 30% of plots are cultivated for Boro and Aman rice in the south-central region of Bangladesh. In the case of soil quality, the highest (55% and 50%) plots have clay-loam for Boro and Aman rice production, followed by sandy-loam and loam soil. Less than 10% of farm plots have clay and sandy soil in both rice seasons in Bangladesh.

## 3 Estimation results and discussion

### 3.1 Hypothesis tests of the inefficiency effect and risk functions

This study conducted several tests of a hypothesis to know whether the chosen SPF model is appropriate to expound on the estimates is presented in Table 2. Firstly, this study performed $H_{0}:\beta_{jk}=0$ that the 2^nd^ order variable's coefficient in the translog model is zero, rejected for both Aman and Boro rice, suggesting that the translog model is more suitable than the Cobb-Douglas model. Secondly, $H_{0}:\psi_{1}=\psi_{2}=....\psi_{13}=0$, which denotes the production risk in inputs and soil type is absent from the Boro and Aman rice production that is also rejected, indicating production risk connected with the choice of inputs in the present data. Thirdly, the null hypothesis also rejected that there are no inefficiency effects in the frontier model $H_{0}:\delta_{0}=\delta_{1}=\ldots .\delta_{15}=0,$ indicating the external factors should be included in the production function. Finally, the external factors do not describe the variation in TE also rejected $H_{0}:\delta_{1}=\delta_{2}=....\delta_{15}=0$, revealing that the joint effects of factors included in the technical inefficiency function are important in explaining the variation of rice production in Bangladesh for both Aman and Boro rice. However, separate effects of some factors may not be significant.

**Table 2:** Specification of the hypothesis test and statistical assumptions.

Hypothesis	Boro rice			Aman rice		
	Test results	Critical value	Decision	Test results	Critical value	Decision
$H_{0}:\beta_{jk}=0$	90.46	47.66	Reject $H_{0}$	65.50	47.66	Reject $H_{0}$
$H_{0}:\psi_{1}=\psi_{2}=...\psi_{13}=0$	37.83	27.03	Reject $H_{0}$	61.23	27.03	Reject $H_{0}$
$H_{0}:\delta_{0}=\delta_{1}=\ldots .\delta_{15}=0$	2970.6	31.35	Reject $H_{0}$	1972.61	31.35	Reject $H_{0}$
$H_{0}:\delta_{1}=\delta_{2}=....\delta_{15}=0$	520.44	29.93	Reject $H_{0}$	300.61	29.93	Reject $H_{0}$

Note: Critical value taken from Table 1 of Kodde and Palm (1986) using 1% significance level.

### 3.2 Translog SFP models estimation

The parameter results of maximum likelihood estimates (MLE) of the translog stochastic frontier production (SPF) for Eqs. (9), (10), and (11) are represented in Table 3 to Table 5. In estimating the SPF, the value of the explanatory variables, $X_{ji}$ (j=1,…,7), are divided by their respective mean, and hence the coefficients $\beta_{j}$ of $lnX_{ji}$ (j=1,…,7) can be interpreted as output elasticities of the corresponding inputs evaluated at their means. All estimated coefficients of the first-order SPF model are between zero to one, meaning that monotonicity conditions are satisfied and all the marginal products are positive and diminishing at the mean of output. The output elasticity of land is the highest 0.86 and 0.76 for Boro and Aman rice in Bangladesh, respectively. This result is consistent with Azad and Rahman (2017) found that the output elasticity of land is much higher than other inputs that are 0.97 and 0.71, respectively. The elasticity of labor is 0.084 for Aman rice, denoting that 8.4% of Aman rice production will be increased by adding 1% more labor supply. Although, output elasticity for labor is insignificant for Boro rice, indicating labor abundance in Boro season. The elasticity of seed is 0.040 and 0.024 for Boro and Aman rice indicating Boro and Aman rice production will be increased 4.0% and 2.4% by increasing 1% more seeds, respectively. Moreover, the contribution of other inputs cost, including irrigation and rental cost of mechanical power, is significant for Boro rice production. Azad and Rahman (2017) argued that increased investment in irrigation accelerates rice productivity, and the rental cost of mechanical power can promote rice productivity. However, farm assets will give a tiny contribution to Boro rice production.

**Table 3:** MLE for parameters of the translog SFP models.

Variable	Boro rice	Aman rice
Land	0.856 (0.018) ∗∗∗	0.764 (0.022)∗∗∗
Labor	0.018 (0.013)	0.084 (0.018)∗∗∗
Fertilizer	0.026 (0.009) ∗∗∗	0.101 (0.012)∗∗∗
Seed	0.040 (0.010)∗∗∗	0.024 (0.014)∗
Pesticide	0.007 (0.006)	0.032 (0.008)∗∗∗
Other ​inputs	0.055 (0.009) ∗∗∗	0.016 (0.012)
Farm ​capital	0.009 (0.003) ∗∗∗	0.005 (0.005)
Land×land	-0.027 (0.028)	-0.023 (0.019)
Labor×labor	-0.006 (0.013)	-0.013 (0.011)
Fertilizer×fertilizer	0.029 (0.007)∗∗∗	0.018 (0.006)∗∗∗
Seed×seed	-0.006 (0.011)	0.003 (0.011)
Pesticide×pesticide	0.006 (0.003)∗∗	0.006 (0.004)
Other ​inputs×other ​inputs ​ ​ ​	0.006 (0.003)∗∗	0.003 (0.001)∗∗
Farm ​capital×farm ​capital	0.0003 (0.001)	0.0003 (0.001)
Land×labor	- 0.038 (0.034)	0.076 (0.025)∗∗∗
Land×fertilizer	-0.032 (0.022)	-0.009 (0.016)
Land×seed	0.006 (0.028)	0.008 (0.024)
Land×pesticide	0.038 (0.013)∗∗∗	0.010 (0.013)
land×other ​inputs	0.052 (0.024)∗∗	-0.037 (0.016)∗∗
land×farm ​capital	0.003 (0.006)	-0.004 (0.006)
Labor×fertilizer	0.005 (0.019)	-0.059 (0.016)∗∗∗
Labor×seed	0.064 (0.019)∗∗∗	-0.012 (0.020)
Labor×pesticide	-0.013 (0.011)	0.006 (0.013)
Labor×other ​inputs	0.004 (0.018)	0.011 (0.0150)
Labor×farm ​capital	-0.005 (0.005)	0.006 (0.005)
Fertilizer×seed	-0.005 (0.015)	0.014 (0.011)
Fertilizer×pesticide	-0.009 (0.008)	-0.004 (0.007)
Fertilizer×other ​inputs	-0.020 (0.012)	0.019 (0.008)∗∗
Fertilizer×farm ​capital ​	-0.004 (0.004)	0.003 (0.003)
Seed×pesticide	-0.017 (0.008)∗∗	-0.018 (0.009)∗∗
Seed×other ​inputs	-0.023 (0.015)	0.030 (0.011)∗∗∗
Seed×farm ​capital	0.010 (0.004)∗∗∗	0.006 (0.004)
Pesticide×other ​inputs	-0.004 (0.008)	-0.009 (0.007)
Pesticide×farm ​capital	-0.004 (0.002)	-0.008 (0.003)∗∗∗
Other ​inputs×farm ​capital ​	0.004 (0.003)	-0.001 (0.003)
HYV	------	0.326 (0.017)∗∗∗
Hybrid	0.198 (0.010)∗∗∗	0.336 (0.025)∗∗∗
Ground water	-0.081 (0.035)∗∗	0.015 (0.010)
Surface water	-0.102 (0.034)∗∗∗	-0.034 (0.021)∗
Constant	6.834 (0.035)∗∗∗	6.204 (0.020)∗∗∗
$\alpha_{i}$	-0.078 (0.006)∗∗∗	0.141 (0.007)∗∗∗
Return to scale	1.01	1.03
Log likelihood	-78.79	-1579.82

Note: The value in parentheses denote standard errors and "∗, ∗∗, ∗∗∗" denote the significance level at 10%, 5%, and 1%.

The average returns to scale (RTS) for Boro and Aman rice is 1.01 and 1.03, respectively, indicating that the rice farm in both Aman and Boro rice farms function under increasing RTS. This result represents that keeping all other factors are constant if 1% jointly increases for all inputs, Boro and Aman rice production will increase by 1.01%, and 1.03%, respectively, which is consistent with Yang et al. (2016). However, Villano and Fleming (2006) reported decreasing returns to the scale of rice production in Bangladesh and central Luzon, Philippines, respectively. Moreover, Table 3 represents that the square term of fertilizer and other inputs are positively significant in both Boro and Aman rice production, indicating those variables have a significant role and extended further by more and more practice for rice production.

Furthermore, compared with rain-fed as a water source, groundwater and surface water have a negative effect on Boro rice. Given the production of boro rice, it is grown mostly with groundwater irrigation (see Table 2), and it is produced in the dry winter season, while production of Aman rice mostly depends on rainfall and is produced in the rainy season with abundant rainfall. The unexpected abundant rainfall over groundwater irrigation in the crucial time like planting time could negatively affect Boro rice production. In this case, we naturally find the negative effect of groundwater and surface water irrigation and show its disadvantage to rainfall water use. HYV and hybrid varieties produce 33% and 34% higher yields than local varieties for Aman rice, while 20% higher yields than HYV for Boro rice production^8^.

### 3.3 Estimation results for inefficiency model

The estimated coefficients of the explanatory variables of the inefficiency effect model are presented in Table 4. The results show that small and medium farms are positively associated with technical inefficiency for Boro rice production. This result indicates that increasing the small and medium farms will decrease the technical efficiency (TE) for Boro rice. This is because smaller farms are less capable of obtaining economies of scale. Smallholders have several other social, economic, financial, and institutional constraints to adopting modern technology and adequate inputs management. Chang and Wen (2011) found a similar sign in their result. On the other hand, the small and medium farms are significantly positive associated with TE for Aman rice production, indicating small and medium farms increase TE while large farms significantly decrease TE. The farmers and small and medium farms manage their farm practice easily because of less required capital, flexible inputs, and efficiently use of their inputs. This finding is in line with Kagin et al. (2016). The estimated coefficient of gender dummy is significantly negative with inefficiency for Aman rice, indicating that male-headed farm households operate more efficiently than female-headed households. The female household head does not have much time to spend on farming activities that might hamper their rice production efficiency. The coefficient of education is significant and positively associated with efficiency for both Boro and Aman rice production, indicating that the technical efficiency increases with the increase in the education level of farmers. Educated farmers may have better management capacity, more investors to adopt new technology, and efficiently use their rice production resources. The coefficient of off-farm income implies that higher off-farm income increase TE for both Boro and Aman rice production. The off-farm income could reduce financial constraints and enable them to purchase flexible input for their rice production.

**Table 4:** ML estimates for parameters of the inefficiency effects models.

Variable	Boro rice	Aman rice
Marginal farm	-0.006 (0.105)	-0.145 (0.100)
Small farm	0.094 (0.046)∗∗	-0.114 (0.043)∗∗
Medium farm	0.092 (0.026)∗∗∗	-0.075 (0.024)∗∗∗
Large farm	0.009 (0.015)	0.041 (0.014)∗∗∗
Head male	0.039 (0.126)	-0.525 (0.115)∗∗∗
Head's education	-0.023 (0.006)∗∗∗	-0.010 (0.006)∗
Family size	0.076 (0.011)∗∗∗	0.054 (0.012)∗∗∗
Off-farm income	-0.154 (0.026)∗∗∗	-0.070 (0.022)∗∗∗
South eastern	0.480 (0.162)∗∗∗	0.078 (0.124)
Northern part of north	-0.273 (0.139)∗∗	-0.394 (0.134)∗∗∗
North western	-0.711 (0.125)∗∗∗	-0.495 (0.128)∗∗∗
Western	-1.997 (0.139)∗∗∗	-0.770 (0.136)∗∗∗
South western	-0.971 (0.126)∗∗∗	-0.509 (0.121)∗∗∗
South central	-0.408 (0.108)∗∗∗	0.119 (0.112)
Year 2018	0.161 (0.050)∗∗∗	-0.312 (0.051)∗∗∗
Constant	-0.098 (0.297)	0.073 (0.256)

Note: Standard errors are in parentheses and "∗, ∗∗, ∗∗∗" represents the significance level at 10%, 5%, and 1%.

**Table 5:** MLE for the linear production risk function.

Variable	Boro rice	Aman rice
Land	0.944 (0.337)∗∗∗	0.849 (0.279)∗∗∗
Labor	-0.577 (0.260)∗∗	-1.176 (0.241)∗∗∗
Fertilizer	-0.220 (0.087)∗∗∗	0.233 (0.082)∗∗∗
Seed	-0.326 (0.193)∗	0.321 (0.189)∗
Pesticide	-0.012 (0.018)	-0. 569 (0.129)∗∗∗
Other input	-0.132 (0.182)	0.029 (0.151)
Farm asset	-0.241 (0.050)∗∗∗	-0.039 (0.058)
Annual mean temperature	0.795 (0.381)∗∗	0.351 (0.330)
Annual mean rainfall	1.058 (0.818)	-1.095 (0.940)
Soil type: Sandy	0.426 (0.812)	-0.242 (0.611)
Loam soil	0.635 (0.632)	-0.415 (0.503)
Clay-loam	0.247 (0.616)	-0.656 (0.470)
Sandy-loam	0.258 (0.638)	-0.080 (0.466)
Constant	-30.888 (13.183)∗∗	-7.944 (12.316)

Note: Standard errors are in parentheses and "∗, ∗∗, ∗∗∗" denote significance level at 10%, 5%, and 1%.

All but except the south-eastern zone are significantly positively influenced TE compared to the northeastern zone. This result indicates that the farmers in these regions are more attentive to farm production and have limited engagement in non-farm activities. Another cause may be that extension services are more active in these zones, and farmers are more attentive to rice production. The coefficient for the year dummy captured a time-varying efficient effect that was found positive and significant for Boro rice, suggesting that farmers produced lower efficiency in 2018 than in 2015. This may be in Boro rice production, farmers cannot improve production risk, or farmers do not adopt new farming methods and technology. The year dummy for Aman rice season is significantly negative, indicating that farmers are more efficient in 2018 than in 2015. This result indicates that the TE has enhanced over the years, possibly due to reducing production risk by improving farming techniques and adopting new farming technology.

### 3.4 Estimation results for risk model

The estimated coefficient of the production risk function is presented in Table 5. The negative and significant coefficient of labor, fertilizer, seed, and farm asset implies that increasing these inputs variable lead to decreased production risk for Boro rice production. This decreasing production risk could make the yield variability of Boro rice production, which is in line with Lemessa et al. (2017). However, the land areas for Boro rice cultivation lead to significantly increased yield variability for Boro rice production. The increasing area under rice cultivation (land) might increase the production risk of exposure of the crops to unexpected climate conditions, especially during the Boro season (dry season). These findings is in line with Tiedemann and Latacz-Lohmann (2013), who argued that larger farms are less capable of responding to unfavorable climate conditions at rice planting or harvesting times. However, Picazo-Tadeo and Wall (2011) explained that land is a risk reduction factor because the rice farmers had fragmented their land into plots so that gains in another compensate for losses from one plot to another due to differences in climatic conditions. Moreover, farm capital significantly reduces the Boro season's yield variability or production risk. This result indicates that the investment in farm capital, especially machinery and equipment, will decrease the production risk of rice in Bangladesh. This result is consistent with the findings of Chang and Wen (2011).

On the other hand, the negative and significant coefficient of labor and pesticide indicates that applying this input variable leads to decreased production risk and yield variability for Aman rice. Table 5 shows that increasing labor in farm practice decreases production risk in Aman rice. This result is consistent with Wang et al. (2020). The positive and significant coefficient of fertilizer denotes that an increase in fertilizer will increases production risk. This is because the farmer does not know the recommended doses of fertilizer, or they apply inefficiently. The coefficient of seed is found to have a risk-increasing effect on Aman rice production, which could cause increased yield variability. These findings are consistent with the results of Oppong et al. (2016), Yang et al. (2016), and Picazo-Tadeo and Wall (2011).

Better soil quality can supply higher nutrients to rice production and hence attain more stable rice production than poor quality, reducing yield variability. However, this study does not find significant value compared to sandy soil. Yang et al. (2016) represent that better soil quality reduces the production risk of rice in China. Moreover, the average monthly temperature was found to have a significantly risk-increasing effect on Boro rice production. Boro rice is produced during the winter dry season, and in this case, increased temperature is harmful for Boro rice production. However, this study did not find any significant rainfall effect on Aman rice production. This finding is consistent with Chang and Wen (2011) argued that temperature is a significantly risk-increasing effect, but rainfall had no significant effect on yield variability.

### 3.5 Distributions of production risk and technical efficiency

**Figure 2:**
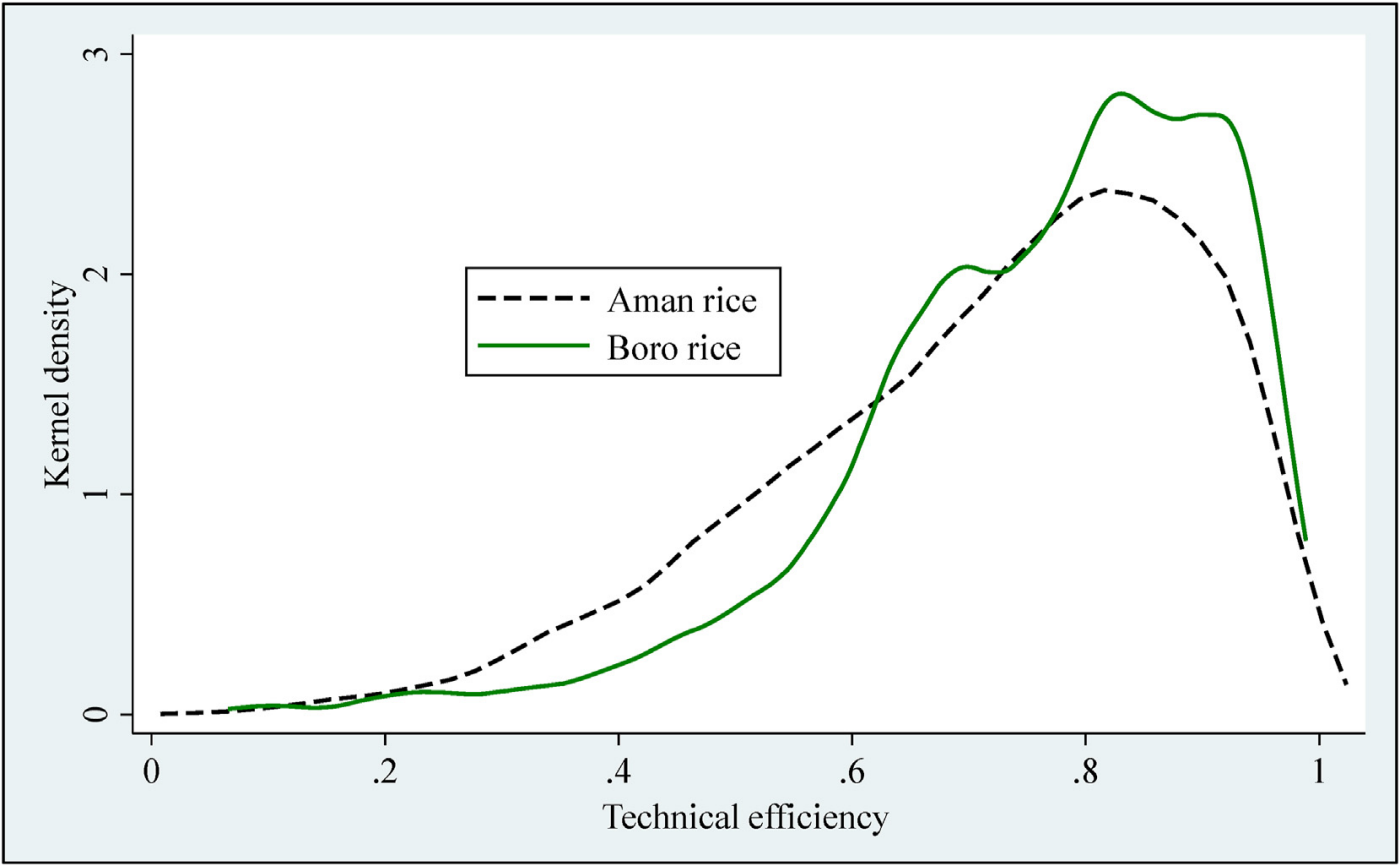
Distribution of TE for Aman and Boro rice.

Table 6 represents the distribution of technical efficiency (TE) and production risk (PR) of Boro and Aman rice in Bangladesh. The average TE score of Boro and Aman rice is 76% and 72%, respectively, indicating a 24% and 28% higher output could be attained by improving technical management, good agronomic practices and improved technologies without increasing existing resources. This difference in the results is because farmers choose and distribute input bundles differently between the season and face various issues or challenges in which farmers operate within these production seasons. The findings that the TE of all farm categories except marginal farms decreased in 2018 than 2015 for Boro rice production. This could be the cause of unfavorable weather conditions for Boro rice production. This could be small, medium and large farmers have not been able to reduce risk with appropriate crop management. However, the marginal farms increased TE in 2018 than 2015 because marginal farms could decrease the risk from natural calamities, increase crop diversification, labor allocation over farming seasons, and efficient use of resources. All farm categories increased TE in 2018 compared with 2015 for Aman rice production due to better farm management, efficient use of resources etc. However, large farms were the highest technically efficient in 2015, while medium farms were the highest technically efficient in 2018 for Aman rice production. The result shows that in both Aman and Boro rice production, farmers reduced their production risk in 2018 than in 2015. Overall, large farms are more technically efficient than other farms for both Aman and Boro rice production because large farms can use more technology, especially farm mechanization. Figure 2 represents the visual inspection of the difference in technical efficiency.

**Table 6:** Distribution of mean TE and production risk by farm categories.

Technical efficiency						
	Boro season			Aman season		
	2015	2018	All	2015	2018	All
landless	0.7783	0.7714	0.7749	0.6959	0.7099	0.7023
Marginal	0.7595	0.7738	0.7668	0.7035	0.7198	0.7117
Small	0.7592	0.7514	0.7554	0.6910	0.7291	0.7102
Medium	0.7615	0.7440	0.7522	0.7161	0.7486	0.7329
Large	0.8084	0.7718	0.7928	0.7336	0.7468	0.7393
All	0.7642	0.7574	0.7608	0.7012	0.7307	0.7160
Production risk						
landless	0.0179	0.0165	0.0172	0.0250	0.0231	0.0241
Marginal	0.0195	0.0180	0.0187	0.0248	0.0230	0.0239
Small	0.0189	0.0174	0.0182	0.0255	0.0233	0.0244
Medium	0.0212	0.0199	0.0205	0.0277	0.0253	0.0265
Large	0.0211	0.0214	0.0213	0.0325	0.0295	0.0312
All	0.0195	0.0181	0.0188	0.0261	0.0239	0.0249

Source: Author's calculations.

Moreover, the average risk value is 0.0188 and 0.0249 for the Boro and Aman rice seasons, respectively, indicating that the farmer faces a higher production risk in the Aman season in Bangladesh. This might cause the Aman rice to depend more on environmental conditions, especially irrigation, and yield variability could be increased if the farmer faced adverse weather. We also found that for all farm categories for Boro and Aman, production risk in 2015 was higher than in 2018 in Bangladesh.

## 4 Conclusions

The stochastic production frontier has been estimated assuming a true random effect (TRE) approach, including flexible risk features to investigate efficiency, production risk, and the determinants of rice production using seasonal balanced panel data on 5088 plots and 5638 plots for Boro and Aman rice in Bangladesh, respectively. This article investigates and compares the production risk, and technical efficiency (TE) for Boro and Aman rice due to the use of inputs level, technology, environmental condition, etc. are different from each other (Table 1). The estimated elasticities of cultivated area, fertilizer, and seed costs positively and reassert influence Boro and Aman rice production in Bangladesh. The finding from the return to scale revealed that rice producers in Bangladesh exhibit increasing returns to scale in both Boro and Aman, indicating rice producers operate in stage one of the production techniques, meaning that increase in the size of operation to take advantage of economies of scale.

Among the input variables, labor, fertilizer, seed, and farm capital have a risk-reducing, whereas cultivated rice area and mean temperature have a significant risk-increasing effect on Boro rice production. Since Boro rice is a winter crop, the increasing temperature might cause yield variation. In contrast, fertilizer and seed is significantly risk-increasing, whereas labor and pesticide have a significantly risk-reducing effect on Aman rice production. This is because the farmers do not follow the recommended doses for fertilizer. Moreover, this study found that the average risk decreases over the year for Bangladesh's Boro and Aman rice production. The Boro and Aman rice producers produce 76% and 72% of the probable frontier production, given the existing technology and input levels while considering production risk or variance. These findings suggest that 24% and 28% of probable output is lost due to technical inefficiency and production risk for Boro and Aman rice, respectively. The large farm is the highest technically efficient for rice production. The estimated inefficiency model shows that small farms, medium farms and family sizes are significantly and negatively while education and off-farm income are significantly and positively associated with TE for Boro rice. In contrast, small farms, medium farms, education and off-farm income are significantly and positively associated with TE for Aman rice production. Moreover, technical efficiency declined for the Boro rice season while improved over time for Aman rice production.

Finally, the rice yield can be enhanced by choosing an appropriate input bundle based on the rice-growing season (Boro and Aman), and risk-mitigation measures need to be put in place for farmers on how to reduce the risk associated with the use of the factor of production and the environmental risk through training and extension services of the farmers. In this study, policies should focus on improving research and more information to farmers for improvement and management practice in both rice production. Another policy should give more attention to the soil improvement program where the soil research institute should come forward, providing more information to farmers on improving soil quality to achieve stable production by reducing production variability. The government of Bangladesh can emphasize more investment in research to develop advanced climate zone-specific technology and provide input subsidies that have been found to reduce yield variability. Additionally, reducing rice yield variability will require policies that improve farmers' access to extension services to encourage the adoption of climate zone-specific technologies and appropriate crop management practices.

## Declarations

### Author contribution statement

Md Abdus Salam: Conceived and designed the experiments; Performed the experiments; Analyzed and interpreted the data; Contributed reagents, materials, analysis tools or data; Wrote the paper.

### Funding statement

This research did not receive any specific grant from funding agencies in the public, commercial, or not-for-profit sectors.

### Data availability statement

Data will be made available on request.

### Declaration of interest's statement

The authors declare no conflict of interest.

### Additional information

No additional information is available for this paper.

## Footnotes

1 This study did not consider Aus rice production due to small number of observations.

2 Quantity of both family and hired labor.

3 This study consider urea, triple super phosphate, single super phosphate, mono-ammonium and di-ammonium phosphate, muriate of potash and gypsum. The author does not include npks and lime/calcium due to large number of missing data.

4 Total cost of pesticide, insecticide and herbicide.

5 Farm capital is calculated as household level current value of farm assets.

6 I did not consider Aus rice production in this analysis due to small number of observations.

7 Following Department of Agricultural Extension (DAE) the farmers were classified as: landless (<0.5 acre of land), marginal (0.51–1.0 acres of land), small (1.01–2.50 acres of land), medium (2.51–5.0 acres of land) and large (>5.01 acres of land).

8 Farmers did not cultivate local variety in Boro rice season (See Table 1).
